# The pharyngeal taste organ of a blood-feeding insect functions in food recognition

**DOI:** 10.1186/s12915-024-01861-w

**Published:** 2024-03-13

**Authors:** Isabel Ortega-Insaurralde, José Manuel Latorre-Estivalis, Andre Luis Costa-da-Silva, Agustina Cano, Teresita C. Insausti, Héctor Salas Morales, Gina Pontes, Martín Berón de Astrada, Sheila Ons, Matthew DeGennaro, Romina B. Barrozo

**Affiliations:** 1 Laboratorio de Neuroetología de Insectos, Departamento Biodiversidad y Biología Experimental (DBBE), Instituto Biodiversidad Biología Experimental y Aplicada (IBBEA), CONICET, Facultad Ciencias Exactas y Naturales, Universidad de Buenos Aires Buenos Aires, Argentina; 2 Laboratorio de Insectos Sociales, Instituto de Fisiología Biología Molecular y Neurociencias (IFIBYNE), CONICET, Universidad de Buenos Aires, Buenos Aires, Argentina; 3 Department of Biological Sciences and Biomolecular Sciences Institute, Florida International University, Miami, FL USA; 4 Laboratorio de Fisiología de la Visión, Departamento de Fisiología Biología Molecular y Celular (FBMC), Instituto de Biociencias Biotecnología y Biología Traslacional (IB3), Facultad Ciencias Exactas y Naturales, Universidad de Buenos Aires, Buenos Aires, Argentina; 5 Université François Rabelais, Tours, France; 6 Laboratorio de Neurobiología de Insectos, Facultad de Ciencias Exactas (CENEXA), Centro Regional de Estudios Genómicos, CONICET, Universidad Nacional de La Plata, La Plata, Buenos Aires, Argentina; 7 Laboratorio de Ecofisiología de Insectos, Departamento Biodiversidad y Biología Experimental (DBBE), Instituto Biodiversidad Biología Experimental y Aplicada (IBBEA), CONICET, Facultad Ciencias Exactas y Naturales, Universidad de Buenos Aires, Buenos Aires, Argentina

**Keywords:** Taste sense, Hematophagous, PPKs, RNA-seq, ATP

## Abstract

**Background:**

Obligate blood-feeding insects obtain the nutrients and water necessary to ensure survival from the vertebrate blood. The internal taste sensilla, situated in the pharynx, evaluate the suitability of the ingested food. Here, through multiple approaches, we characterized the pharyngeal organ (PO) of the hematophagous kissing bug *Rhodnius prolixus* to determine its role in food assessment. The PO, located antero-dorsally in the pharynx, comprises eight taste sensilla that become bathed with the incoming blood.

**Results:**

We showed that these taste sensilla house gustatory receptor neurons projecting their axons through the labral nerves to reach the subesophageal zone in the brain. We found that these neurons are electrically activated by relevant appetitive and aversive gustatory stimuli such as NaCl, ATP, and caffeine. Using RNA-Seq, we examined the expression of sensory-related gene families in the PO. We identified gustatory receptors, ionotropic receptors, transient receptor potential channels, pickpocket channels, opsins, takeouts, neuropeptide precursors, neuropeptide receptors, and biogenic amine receptors. RNA interference assays demonstrated that the salt-related pickpocket channel *Rproppk014276* is required during feeding of an appetitive solution of NaCl and ATP.

**Conclusions:**

We provide evidence of the role of the pharyngeal organ in food evaluation. This work shows a comprehensive characterization of a pharyngeal taste organ in a hematophagous insect.

**Supplementary Information:**

The online version contains supplementary material available at 10.1186/s12915-024-01861-w.

## Background

Contact chemoreception or taste is the sensory modality that allows an animal to detect non-volatile chemicals on a substrate or in a liquid medium. Among other functions, such as identifying conspecifics and/or oviposition sites, the taste system allows animals to predict the nutritional or toxic quality of the food even before feeding on it. Thus, the taste sense drives the final feeding decisions of animals that will undoubtedly have determinant physiological consequences in their lives [1].

Food evaluation in insects is a highly relevant task performed by specialized peripheral sensory organs and their functional units, the taste sensilla. At this stage, the presence or absence of chemical components of the food leads to the final decision: to eat or not to eat. Gustatory receptor neurons (GRNs) represent the cellular platform of the taste system housed within the taste sensilla [2]. Embedded in the membranes of GRNs, sensory protein receptors are responsible for the molecular detection of taste stimuli. The activation of these GRNs evokes an electrical signal that travels to the insect brain, triggering the decision to accept or reject the food.

A sequence of feeding events starts once a blood-feeding insect reaches a vertebrate host, as has been described for the kissing bug *Rhodnius prolixus* [3]. Initially, the blood feeder carries out an external gustatory assessment of the host skin by using the antennal taste sensilla [4, 5], although tarsal taste sensilla cannot be ruled out to play a role in the skin taste assessment. No taste sensilla was identified in the proboscis of kissing bugs [6, 7]. If no aversive stimuli are detected on the skin, the insect pierces it [4, 5]. Second, before starting the true ingestion, pharyngeal and cibarial muscles located in the head produce contractions, sucking a small quantity of blood. The insect does an internal gustatory evaluation of the incoming food [8]. If the ingested blood fulfills the insect’s feeding requirements, the animal continues the feeding behavior; if not, the animal leaves the host and searches for another [3, 9]. The gustatory blood assessment seems to occur in internal taste sensilla located strategically along the pharynx*.* The eight short-peg sensilla that forms the pharyngeal organ (PO) of kissing bugs appear as candidates to accomplish this function [4, 7, 10]. Similar internal sensilla or taste papillae are described on the cibarium of *Aedes aegypti* mosquitoes and species of the genera *Culex* sp., *Culiseta* sp., and *Anopheles sp*., the labro-cibarial sensilla of *Simulium *spp. [11–14]. All these sensilla are bathed with blood as soon as the first sip passes through the pharynx.

Few chemicals present in the ingested blood have been shown to induce blood feeding in insects, and the identification of these components could lead to the development of anti-feedants [3]. Adenosine nucleotides are the main known phagostimulant compounds in the vertebrate blood. They are released by lysis of erythrocytes, platelets, and epithelial cells as a damage response to the piercing or severing during blood intake [15–17]. Several insects are responsive to ATP, including kissing bugs, some mosquitoes, tsetse flies, and fleas, while others respond better to ADP [3]. Low concentrations of salts, necessary to maintain the endogenous salt balance, have also been proved to elicit appetitive behaviors in kissing bugs, mosquitoes, fleas, and bed bugs [8, 18–22]. On the other hand, molecules perceived as bitter by humans like quinine, caffeine, and quinidine have an anti-feedant effect in mosquitoes such as *An. gambiae* and *Ae. aegypti* [23–26]. Furthermore, the gustatory detection in the feeding solution of quinine, caffeine, berberine, salicin, or high NaCl (> 0.2 M) inhibits feeding in *R. prolixus,* even if it also contains appetitive stimuli such as ATP and appetitive salt doses [5, 8, 27, 28].

*Rhodnius prolixus* is a competent vector of the Chagas disease that transmits the protozoan parasite *Trypanosoma cruzi* to humans through its feces following a blood meal. This neglected parasitic disease affects several countries in Latin America (WHO, 2020). Under this context, taste perception represents a relevant target for suppressing blood feeding and, subsequently, pathogen transmission. Morphological studies have postulated the PO of *R. prolixus* as the blood food sensor [4, 7, 10]. However, the sensory basis of blood gustatory assessment by internal sensory organs has been barely studied in *R. prolixus* and in blood-feeding insects in general [3, 29]. In this work, our objective was to provide neuroanatomical, physiological, and molecular evidence uncovering the gustatory role of the PO of *R. prolixus* in food assessment through a multi-approach analysis. We identified gustatory receptor neurons (PO-GRNs) in taste sensilla of the PO, which respond to gustatory stimuli relevant to kissing bugs, such as NaCl, ATP, and caffeine. The taste information then reaches the subesophageal zone in the brain via the labral nerves, where it is likely primarily processed by the CNS, as observed in other insects [30, 31]. Furthermore, the PO expresses a vast repertoire of genes, among which several candidates may be related to food recognition functions. By reducing the expression of one of these genes, the pickpocket channel receptor *Rproppk014276*, feeding of an appetitive solution is prevented. Altogether, we demonstrate the role of the PO in assessing the taste of ingested food in *R. prolixus.*

## Results

### The pharyngeal organ (PO) houses GRNs

**Fig. 1:**
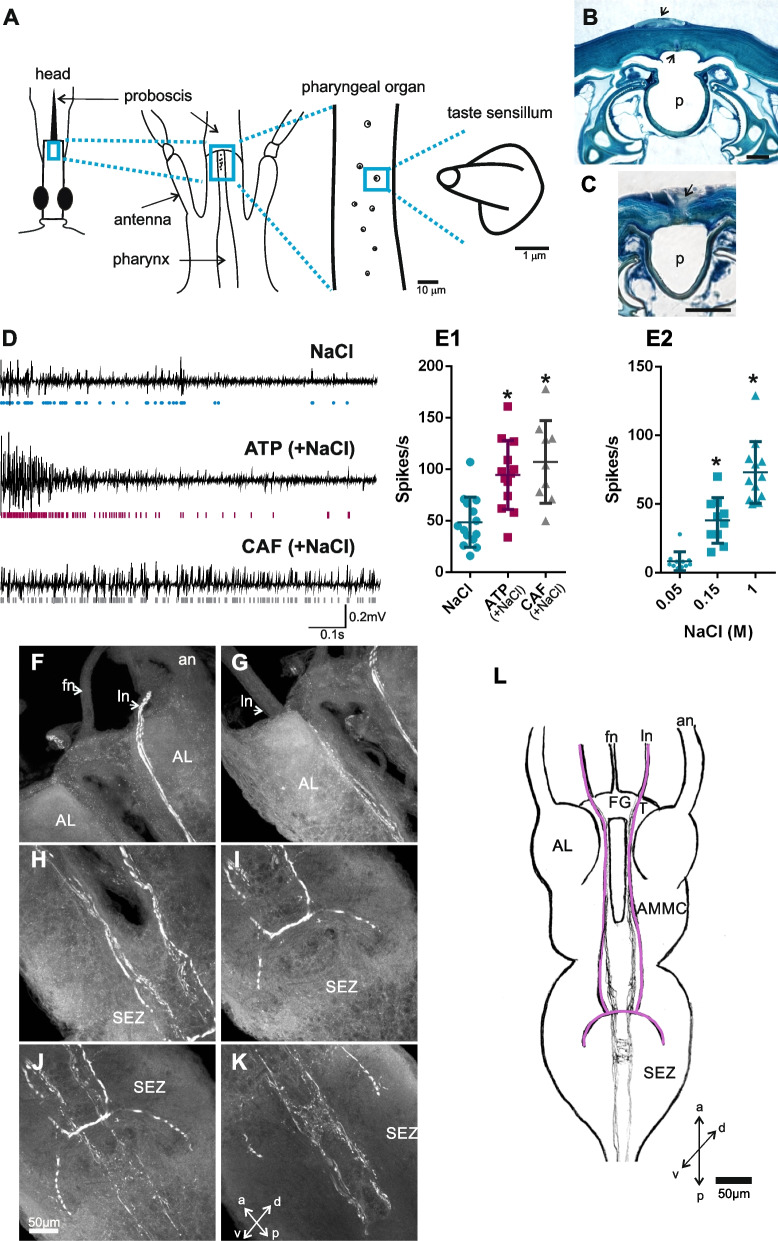
Morphology and function of taste sensilla of the PO. **A** Schemes of the head, the PO, and the single uniporous sensilla at different scales of *R. prolixus*. A longitudinal and ventral view of the head and pharynx, unveiling the eight taste sensilla of the PO. The PO is located antero-dorsally to the head and pharynx. **B–C** Photographs of histological cross sections of the PO. **B** Dorsally to the pharynx (p), indicated with arrows, a taste sensillum and two cell bodies can be identified. Horizontal bar denotes 2 µm. **C** A dendrite of a putative PO-GRN is shown with an arrow. Horizontal bar indicates 10 µm. **D–E** Electrophysiological responses of PO-GRNs. **D** Example of spike discharges of the GRNs of the PO. Typical responses to NaCl, ATP, and caffeine are shown. Circles and bars below traces represent the spike events following spike sorting. **E1–2** Scatter plots are shown, and the horizontal lines represent the mean responses (mean ± s.e.) of the summed spike events of the PO-GRNs upon stimulation. **E1** ATP and caffeine produced significantly more spike events than NaCl. The solutions of ATP and caffeine also contained NaCl (0.15 M), used as control stimulation. Asterisks indicate statistical differences between insects stimulated with ATP or caffeine versus those stimulated with NaCl alone (*p* < 0.0005) (NaCl, *n* = 14; ATP, *n* = 13; caffeine, *n* = 9). **E2** Increasing doses of NaCl elicited a corresponding increase in the firing responses. Asterisks indicate statistical differences between insects stimulated with 0.05 M NaCl versus those stimulated with 0.15 M and 1 M NaCl (*p* < 0.05) (NaCl 0.05 M, *n* = 11; NaCl 0.15 M, *n* = 10; NaCl 1 M, *n* = 12). **F–L** Anterograde staining of the PO-GRNs.** F–K** Photographs at different levels of the brain of insects, showing two subtypes of PO-GRNs reaching the subesophageal zone (SEZ). One subtype constituted by thick axons showed to project contralaterally in the SEZ exclusively (denoted in pink). Another subtype of PO-GRNs (denoted in gray) arborise ipsilaterally at the medial and dorsal region of the SEZ and continue to the posterior ganglions (data not shown). **L** Reconstruction of the arborizations of PO-GRNs that reach the brain. fn frontal nerve, ln labral nerve, an antennal nerve, FG frontal ganglion, T tritocerebrum, AL antennal lobe, AMMC antennal motor mechanosensory center, SEZ subesophageal zone, a anterior, p posterior, d dorsal, v ventral

The PO is the unique sensory organ bathed with the ingested blood in *R. prolixus*. Therefore, the eight short-peg sensilla, located along 80 µm, in the anterodorsal region of the pharynx of *R. prolixus* (Fig. 1A) must be involved in the internal taste evaluation of incoming food. Dispersedly distributed, these uniporous sensilla are candidates to sense the incoming blood meal (Fig. 1A). Histological transverse sections of the pharynx allowed the recognition of winding dendrites inside the sensilla (Fig. 1B, C). Figure 1C shows a dendrite of a sensory neuron, candidate for being a PO gustatory receptor neuron responsible for food sensing.

Key taste components of the ingested food might be detected in the PO-GRNs. Therefore, the functional role of PO-GRNs in food detection was confirmed through electrophysiological recordings (Fig. 1D, E). The electrophysiological activity of these sensory neurons was recorded near the PO and the afferent labral nerves. We recorded the summed extracellular responses of the PO-GRNs transmitted through the labral nerve, upon gustatory stimulation with NaCl, ATP, and caffeine (Fig. 1D, E), three stimuli known to produce appetitive or aversive feeding responses in *R. prolixus*. In all the recordings, neuronal activity was observed for all three stimuli (Fig. 1D, E). Despite several responding neurons being activated, spike sorting of the responses of individual neurons proved challenging. The PO-GRNs showed activation to NaCl, ATP, and caffeine (Fig. 1E1). The overall firing activity to ATP or caffeine was significantly increased over NaCl-elicited responses (Fig. 1E1) (K-W = 15.3, *p* = 0.0005, post hoc Dunn’s comparisons against NaCl control group *p* < 0.05). In Fig. 1E2, we also demonstrate the rise in activity of PO-GRN neurons upon stimulation with increasing doses of NaCl (K-W = 25.9, *p* = 0.0001, post hoc Dunn’s comparisons *p* < 0.05). These results show that the sensilla present on the PO effectively house sensory neurons sensitive to behaviorally relevant stimuli, demonstrating a role for the PO during taste assessment of incoming food in *R. prolixus.*

### The PO-GRNs project into the subesophageal zone

Following stimulus detection, taste information must reach the brain for integration and processing. Therefore, and through anterograde backfills of PO-GRNs, we examined the morphology, topography, and sites of arborizations of these sensory neurons in the central nervous system. Five preparations showed stained neuronal arborizations in the brain. Two morphotypes or subtypes of GRNs were recognized. Axons of these neuronal subtypes extend from the PO via the labral nerves (Fig. 1F–K). A bundle of thin subtypes of PO-GRNs (Fig. 1F–K, denoted in gray in Fig. 1L) arborized ipsilaterally at the medial and dorsal region of the subesophageal zone (SEZ) (Fig. 1J–K) and continue to the posterior ganglions (data not shown). Another subtype of PO-GRN was easily recognized due to its thickness regarding the other subtype (Fig. 1I–J, denoted in pink in Fig. 1L). Interestingly, these subtypes of neurons showed to project contralaterally in the SEZ exclusively, as indicated in the photo (Fig. 1I–J) and the reconstruction (Fig. 1L). These results showed the SEZ as the primary central relay of PO-GRNs in *R. prolixus*.

### The PO expresses several sensory-related gene families

**Fig. 2:**
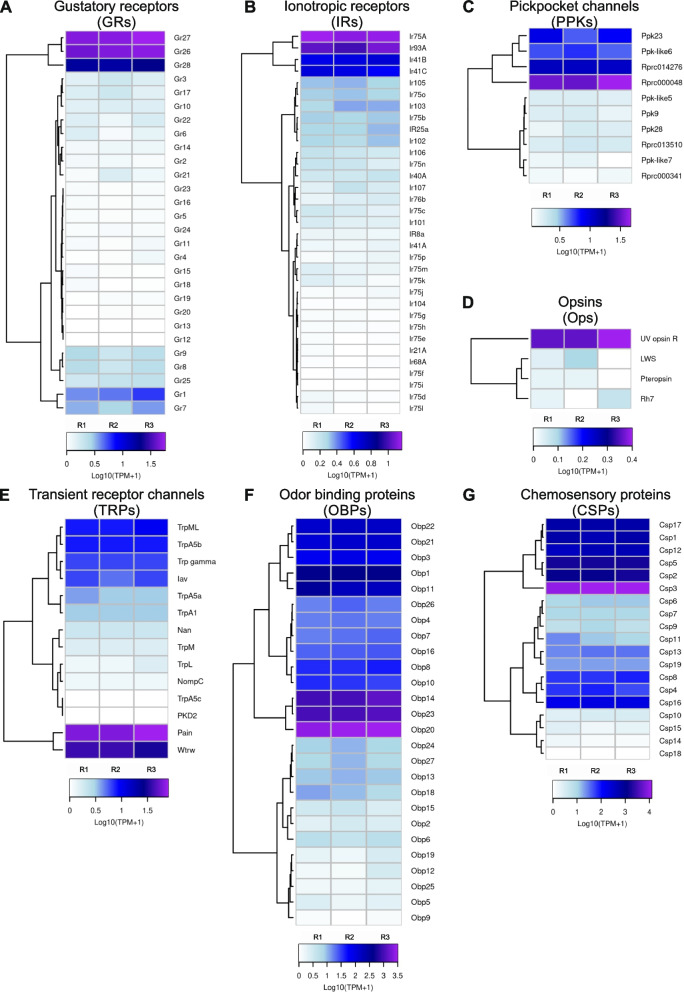
Expression of sensory-related genes in the PO. Heatmaps were built using Log_10_ (transcripts per kilobase per million read (TPM) + 1) as input of the gplot R package. Transcript abundance was represented in a color scale where white/purple represents the lowest/highest expression. A dendrogram was plotted using hierarchical clustering of gene expression values based on Euclidean distances and a complete linkage method for clustering. **A** Gustatory receptors (GRs). **B** Ionotropic receptors (IRs). **C** Pickpocket ion receptor channels (PPKs). **D** Opsins (Ops). **E** Transient receptor potential channels (TRPs). **F** Odorant-binding proteins (OBPs). **G** Chemosensory proteins (CSPs)

**Fig. 3:**
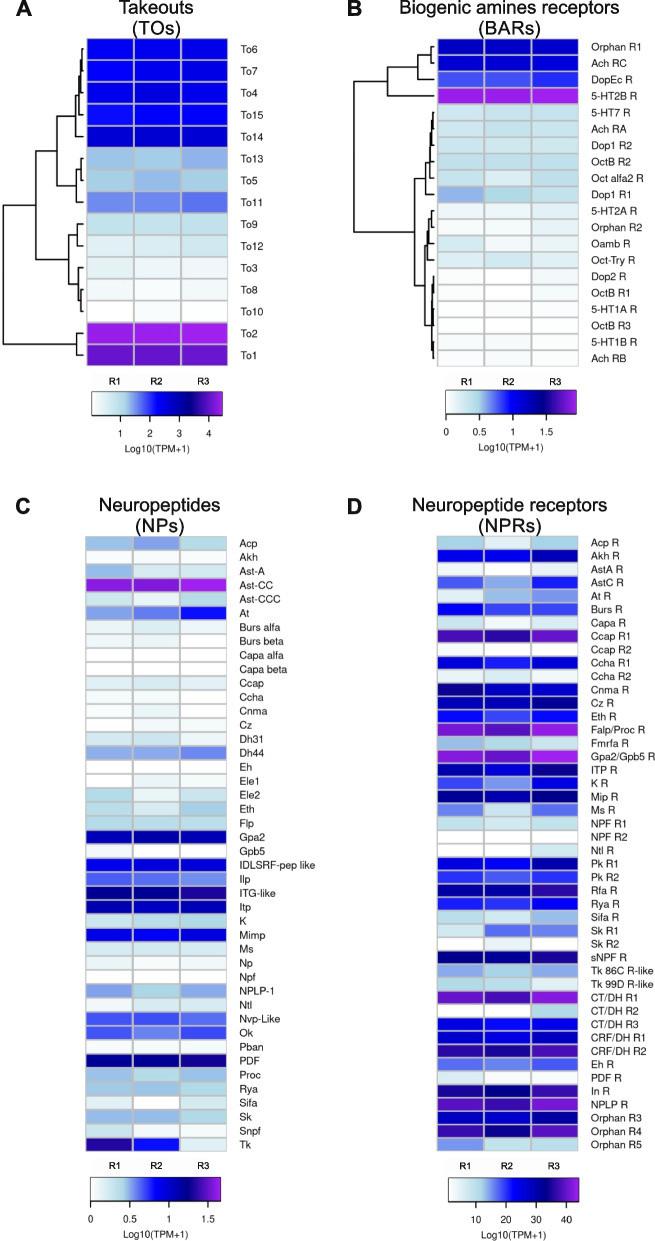
Expression of neuromodulatory genes in the PO. Heatmaps were created using Log_10_ (transcripts per kilobase per million read (TPM) + 1) as input of the gplot R package. Transcript abundance was represented in a color scale where white/purple represents the lowest/highest expression. A dendrogram was plotted using hierarchical clustering of gene expression values based on Euclidean distances and a complete linkage method for clustering. **A** Takeout genes (TOs). **B** Biogenic amine receptors (BARs). **C** Neuropeptides (NPs). **D** Neuropeptides receptors (NPRs)

RNA sequencing (RNA-Seq) allowed us to examine the gene expression of the isolated PO. We found representatives of gene families associated with sensory functions previously characterized in other insects (Fig. 2). Several of them possess putative gustatory functions relevant during food recognition (Figs. 2 and 3). A total of 307,059,020 raw reads were produced from three libraries. After quality control and trimming, a total of 91.1 M, 75.7 M, and 78.9 M of cleaned reads were obtained for each of the three replicates. A total of 53.5 M, 42.4 M, and 49.1 M reads mapped to the *R. prolixus* genome. Gene expression data of the main sensory-related gene families, presented as Log_10_ (TPM (transcripts per kilobase per million read) + 1), were obtained (Figs. 2 and 3). Among others, we found sensory genes representative of seven families that include: gustatory receptors (GRs), ionotropic receptors (IRs), pickpocket channels (PPKs), transient receptor potential channels (TRPs), opsins (Ops), odorant-binding proteins (OBPs), and chemosensory proteins (CSPs) (Fig. 2). Takeouts (TOs), neuropeptide precursors (NPs), neuropeptide receptors (NPRs), and receptors of biogenic amines (BARs) were also detected; many of them were demonstrated to take part in the regulatory mechanisms of the feeding behavior in several insect models [32–35] (Fig. 3).

Overall, a low expression of GRs can be noticed (Fig. 2A). However, a few GR encoding genes showed enhanced expression, such as the *RproGr26, RproGr27*, and *RproGr28*. Besides, *RproGr1*, the orthologous gene of the fructose receptor *DmelGr43a* [36]*,* also showed relatively high expression levels.

Regarding IR expression, high levels for *RproIr93a* and *RproIr75a* were detected (Fig. 2B). Yet, and to a lesser extent, high expression was observed for *RproIr41b* and *RproIr41c* (Fig. 2B). IRs are ligand-gated ion channels derived from variant ionotropic glutamate receptors that can function as chemo-, hygro-, and thermoreceptors [37, 38]. It is feasible that these candidates possess similar roles in *R. prolixus*.

PPKs are a family of amiloride-sensitive degenerin/epithelial sodium channels (DEG/ ENaC) that mediate salt and water sensation, among other functions [39, 40]. Interestingly, from the ten PPK-encoding genes present in the genome of *R. prolixus*, *RPRC000048*, *Rproppk014276*, *Rproppk23,* and *Rproppk-like6,* exhibited high expression in the PO (Fig. 2C). This is similar to what occurs in the antennal sensilla for the *Rproppk014276* [5], this PPK could be an interesting candidate to play a role in salt detection within the PO.

Among the four opsins that were found to be detected in the PO, *RproUVopsin* was the most expressed (Fig. 2D). Opsins are known to be involved in photo-, mechano-, and thermosensation [41]. More recently, these receptors were demonstrated to behold a gustatory function in the *D. melanogaster* labellum [42]. Opsin expression has never been shown before in the gustatory tissue of a blood-sucking insect.

TRPs are a superfamily of cation-conducting membrane proteins involved in mechano-, chemo-, photo-, and thermosensation [43, 44]. Regarding the TRP repertoire expressed in the PO tissue, an enhanced expression of *RproPainless* and *RproWaterwitch* followed by *RproTrpml*, *RproTrpa5b, RproTrp-gamma*, and *RproInactive* were observed (Fig. 2E). This highly conserved sensory gene family might represent putative sensors of thermal, nociceptive, and bitter cues in the alimentary canal.

High expression levels of OBPs-encoding genes in the PO were also identified. The high expression levels of *RproObp20, RproObp14,* and *RproObp23* is noteworthy (Fig. 2F). Moreover, we found high expression of CSP-encoding genes in the PO, especially *RproCsp3, RproCsp5, RproCsp2, RproCsp1, RproCsp17*, and *RproCsp12* (Fig. 2G). OBPs and CSPs are soluble proteins that bind small molecules, such as odorants and pheromones, in the lymph of insect chemosensilla [45] among other functions in non-sensory tissues [46]. The relative enhanced expression of OBPs and CSPs in the PO heavily indicates them as attractive candidates to aid in the mechanisms of food evaluation.

The TOs are circadian regulated genes whose expressions are induced by starvation and act as hormone carriers [32, 47, 48]. TOs are thought to regulate the peripheral sensitivity to phagostimulant stimuli under starvation conditions and male courtship behavior [32, 47, 49–51]. They are usually expressed in organs such as labella, crop, antenna, and brain. We found that TOs encoding transcripts are also detected in the PO. *RproTo2* and *RproTo1* showed the highest expression concerning other members of the same family (*RproTo4*, *RproTo6*, *RproTo7*, *RproTo14,* and *RproTo15*) which also presented high levels of relative expression (Fig. 3A). High expression levels of biogenic amine receptors (BARs) were also detected, particularly the 5-hydroxytryptamine 2B receptor (*Rpro5HTR2b*), the acetylcholine receptor C (*RproAChRc*), and the dopamine/ecdysone receptor (*RproDopEcR*) (Fig. 3B). The *Rpro5HTR2b* is activated by 5-hydroxytryptamine, i.e., serotonin, and *R. prolixus* depends on this serotonergic system to successfully complete the feeding behavior [52]. Interestingly, the presence of the *AChRc* in a gustatory organ has not been shown before in any organism studied so far. *DopEcR* is activated by dopamine and ecdysteroids, such as ecdysone and 20-hydroxyecdysone [53, 54]. Serotonin, dopamine, and 20-hydroxyecdysone are known to control and regulate feeding in insects [54–57].

Transcripts encoding neuropeptide precursors (NPs) and neuropeptide receptors (NPRs) were also identified in the PO; however, their expression can range from low or moderate to high levels (Fig. 3C, D). High expression of both the precursor and receptor was detected only for the heterodimeric glycoprotein hormone *RproGPA2/GPB5*, suggesting a paracrine regulation in the PO (Fig. 3C). High expression of the NP precursor genes allatostatin-CC (*RproAst-CC*), *RproITG-like*, ion transporter peptide (*RproITP*), myoinhibiting peptide (*RproMIP*), *RproDLSRF-like* peptide, and pigment-dispersing factor (*RproPDF*) was found, even though the expression of their receptors is low, pointing to a hormonal release from the PO to act in an/other tissue(s) (Fig. 3D). Conversely, the proctolin receptor (*RproFalp/ProcR*), the calcitonin-like/ diuretic hormone receptor 1 (*RproCT/DHR1*), the neuropeptide-like receptor 1 (*RproNPLPR)*, the crustacean cardioactive peptide receptor 1 (*RproCCAPR1)*, the CRF-like/diuretic hormone receptor 2 (*RproCRF/DhR2*), and the insulin receptor (*RproInR)* are highly expressed in the PO. However, the expression of their precursors encoding their peptide ligands are low (Fig. 3D). This could indicate endocrine responses in the PO to neuropeptides released to the hemolymph from other insect tissues. Several of these NPs and NPRs were previously demonstrated to regulate feeding-related events [58, 59].

### Disruption of a PO sensory gene prevents feeding

We decided to investigate the role of PO in the gustatory evaluation of food. Therefore, if the PO serves as the sensory organ dedicated to evaluate the taste quality of the incoming blood, the alteration of the expression of a sensory gene expressed in the PO will affect the acceptance of food, consequently demonstrating the relevance of the PO in food taste recognition. We focused on *Rproppk014276* and *Rproppk28* due to their known roles in salt detection in the antennae of *R. prolixus* [5].

Knowing that NaCl, at an equivalent amount of the vertebrate plasma, is a primary phagostimulant for *R. prolixus* and crucial for triggering feeding [8, 28], we predicted that the knockdown of one or both PPKs could prevent this response.

RNA interference experiments were carried out to disrupt *Rproppk014276* (VectorBase RPRC014276) and *Rproppk28* (VectorBase RPRC000471) expression. Therefore, the four experimental groups consisted of: uninjected insects, dsRNA-ctrl (control group of dsRNA injection), dsRNA-Rproppk014276, and dsRNA-Rproppk28 injected insects. The RT-qPCR results demonstrated effective knockdown of *Rproppk014276* transcript in both the antennae (Additional file 1: Fig. S1) and in the PO of *R. prolixus* (Additional file 2: Fig. S2). Notably, there was a significant decrease in the transcript levels of *Rproppk28* in the antennae, confirming the success of the RNAi knockdown. However, the expression levels of this gene in the PO were so low that it remained undetectable by qPCR, even in the control sample (dsRNA-ctrl group). These results were not surprising, considering the extremely low TPM values for *Rproppk28* in the PO, as shown in Fig. 2C, which could explain these results.

**Fig. 4:**
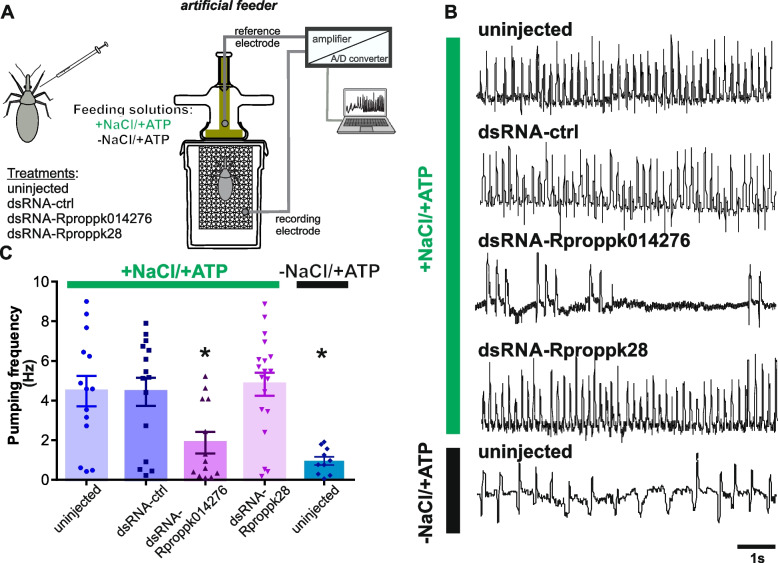
Role of salt-related PPKs during feeding. **A** Experimental design for feeding experiments. The feeding activity of four groups of insects was tested in the artificial feeder: uninjected, dsRNA-ctrl, dsRNA-Rproppk014276, and dsRNA-Rproppk28. The artificial feeder was connected to an electromyographic recording system, where the recording electrode was linked to a metallic mesh supporting the insect, and the reference electrode was immersed in the feeding solution. Once the insect inserted its mouthparts into the feeder, the electrical circuit closed, generating a base conductance. Changes in the baseline signal were attributed to contractions produced by the sucking muscles when the insect initiated feeding. Recorded signals were amplified, digitized, and stored in a PC. All four experimental groups were offered with a + NaCl/ + ATP solution. A control group of uninjected insects was tested with a − NaCl/ + ATP solution. **B** Example of electromyographic recordings of the muscles involved in feeding. Uninjected, dsRNA-ctrl and dsRNA-Rproppk28 groups exhibited similar responses to the + NaCl/ + ATP solution, showing a regular pumping behavior. In contrast, dsRNA-Rproppk014276 insects, when offered with + NaCl/ + ATP or uninjected insects offered with − NaCl/ + ATP solution, displayed low and scattered pumping activities relative to the other groups. **C** Pumping frequency during feeding of treated and uninjected insects on the + NaCl/ + ATP or − NaCl/ + ATP solutions. No differences were found between uninjected, dsRNA-ctrl and dsRNA-Rproppk28 groups. However, dsRNA-Rproppk014276 insects showed significantly lower frequency values than the other groups. Notably, the dsRNA-Rproppk014276 group offered with the appetitive solution + NaCl/ + ATP showed no differences from uninjected insects fed on − NaCl/ + ATP solution. Scatter plots are displayed, and bars represent the mean pumping frequency (mean ± s.e.m.). Asterisks indicate significant differences of each group to uninjected insects fed on the + NaCl/ + ATP solution (*p* < 0.001) (+ NaCl/ + ATP groups: uninjected, *n* = 14; dsRNA-ctrl, *n* = 15; dsRNA-Rproppk28, *n* = 19; dsRNA-Rproppk014276, *n* = 13; and − NaCl/ + ATP uninjected group, *n* = 10)

The feeding response of control and knockdown insects was tested with an appetitive solution composed of NaCl and ATP (+ NaCl/ + ATP). Note that uninjected kissing bugs need both NaCl and ATP to elicit a full appetitive response [8]. Using an artificial feeder coupled to an electromyographic recording system (Fig. 4A), we recorded the activity of the muscles associated with feeding in all experimental groups. Additionally, a group of uninjected insects was offered a feeding solution containing only ATP but not NaCl (-NaCl/ + ATP in Fig. 4). The rationale behind this assay was to show that kissing bugs do not feed on a salt-free solution, even in the presence of ATP. Therefore, if *Rproppk014276* and/or *Rproppk28* are involved in salt detection, we would expect that, after knockdown, bugs would behave similarly to uninjected insects offered a salt-free solution.

The qualitative and quantitative analysis of the electromyograms (EMGs) revealed different feeding patterns based on the treatment and feeding solution provided to each group (Fig. 4B, C). The pumping frequency of the sucking muscles (Fig. 4B, C) and the weight gain (measured as a feeding factor) (Additional file 3: Fig. S3) were analyzed among the group of insects. Regular and highly frequent pumping pulses, where each pump pulse represents a stroke of fluid being pumped by the muscles, were observed in the uninjected and unrelated control (dsRNA-ctrl) groups, as well as the dsRNA-Rproppk28 group. Moreover, insects of these three groups were fully engorged (Additional file 3: Fig. S3).

In contrast, the dsRNA-Rproppk014276 group exhibited fewer pumping pulses (Fig. 4B, C) (K-W = 19.6, *p* = 0.0006, Dunn’s post hoc comparisons against the uninjected group, *p* < 0.05), and insects did not fully engorge by the end of the experiment (Additional file 3: Fig. S3). Notably, a comparable and non-significant difference in the feeding performance was observed between uninjected insects offered a solution lacking NaCl (-NaCl/ + ATP solution) and the dsRNA-Rproppk014276 group offered the appetitive + NaCl/ + ATP solution (Fig. 4B, C, Additional file 3: S3).

It is noteworthy that the antennae do not participate in detecting salt during ingestion, as they never make contact with the feeding solution in the artificial feeder, nor do they come into contact with blood while feeding on a live host. As a result, antennectomized insects exhibit no difference in feeding behavior compared to intact insects when provided with an appetitive + NaCl/ + ATP solution (Additional file 4: Fig. S4).

The undetectable expression levels of the *Rproppk28* transcript in the PO cannot rule out the possibility of the lack of effect from the RNAi treatment for *Rproppk28*. On the other hand, we demonstrated that suppression of *Rproppk014276* expression hampered feeding of an appetitive salt solution. Our results indicate that food taste assessment in the PO is crucial to elicit feeding, as feeding is prevented by the disruption of a salt-related gene (*Rproppk014276*).

## Discussion

Pharyngeal taste machinery represents the last instance of food quality evaluation during ingestion. It was demonstrated that external and internal taste inputs are not functionally redundant; instead, both are required to control feeding in both non-blood feeders like *D. melanogaster* [60] and blood feeders like *R. prolixus* [4]. In this work, we characterized the PO, the internal taste organ of *R. prolixus,* through a multi-approach strategy. Thus, we examined different aspects of its neuroanatomy, physiology, and genetics to unveil the role of this gustatory organ. PO sensilla house GRNs that reach the brain through the labral nerves. Gustatory information from PO-GRNs arrives at two regions of the SEZ where it will be primarily processed, although some inputs continue beyond this neuropile. Furthermore, the profile of gene expression in the PO provided molecular candidates likely involved in the gustatory assessment of the incoming blood. The knockdown of a sensory gene expression interferes with the feeding behavior, probably by affecting NaCl detection, one of the main phagostimulants of this species.

### The PO-GRNs are responsible for internal gustatory detection

The histological inspection of the short-peg sensilla on the PO of *R. prolixus* revealed the existence of sensory neurons. The morphology of internal gustatory organs was mostly studied in dipterans. For example, the PO of the fruit fly *D. melanogaster* and their taste sensilla have been deeply examined recently [61]. The cibarial organ of the mosquito *Ae. aegypti* and the tsetse fly *Glossina austeni,* equivalent to the PO in *R. prolixus*, hold taste sensilla which have also been postulated to be sensors of blood components [62, 63]. Although denominated differently, some of the morphotypes of sensilla present in the cibarial organ of both blood feeders are also innervated by chemosensory neurons [62, 63].

We also provided a physiological examination of the PO sensory neurons in a blood-feeding insect. Here, by registering from near the PO and the labral nerves, we demonstrated the neural response to relevant gustatory stimuli for *R. prolixus*. We obtained multicellular responses to two phagostimulants or appetitive compounds, NaCl and ATP, and to one aversive compound, caffeine. NaCl, a main constituent of blood, is necessary to all blood-feeding insects studied so far (e.g., *R. prolixus*, *Triatoma infestans*, *Ae. aegypti*, *Anopheles* spp., *Culex pipiens*, *Culiseta inornata*, *Glossina* spp., *Tabanus nigrovittatus*, *Simulium venustrum*, *Stomoxys calcitrans*, *Xenopsylla cheopis*) to achieve a normal and complete feeding response [18]. Adenosine nucleotides, such as ATP and ADP, released from the erythrocytes and platelets upon shear stress on blood capillaries, also elicit gorging in most blood feeders [3]. In contrast, detection of alkaloids in the ingested food, such as caffeine, quinine, theophylline, and berberine, drives the ultimate decision to reject a meal in *R. prolixus* [1, 4, 27]. Similarly, quinine and caffeine also cause feeding avoidance of sucrose solutions in *Ae. aegypti* [24], while denatonium benzoate and berberine negatively affect sugar feeding in *An. gambiae* [25]. But still, taste detection of aversive molecules in blood feeders is by far insufficient with respect to phytophagous insects [3]. We showed that gustatory evaluation of appetitive and aversive molecules begins in GRNs located in the PO. The fine discrimination of the gustatory information will then occur in the brain, which will ultimately send an output message to the appropriate muscles to avoid ingesting a given resource. Future studies could be aimed at individually characterizing the GRNs of the PO of *R. prolixus*.

### The subesophageal ganglion receives inputs from the PO

Previous morphological studies of pharyngeal neuron projections showed disparate results. In *D. melanogaster*, neurons in the pharynx project exclusively to the SEZ [64]. In turn, *Ae. aegypti* sensory neurons of the cibarium project to the SEZ but also to the tritocerebrum whereas *G. austeni* neurons project exclusively to the tritocerebrum [31, 62, 63]. Our results show that the axons of the PO-GRNs project through the labral nerves primarily into the SEZ, whereas some fibers continue to posterior ganglions. Furthermore, two different types of neurons reached two different regions of the SEZ, showing a differential topography of the PO-GRNs in the brain. This could be the basis of a differential processing of the information that comes from the pharyngeal organ, for example, a sensory segregation of aversive inputs and appetitive as occurs in *Drosophila* [30] and in mammals [65]. However, this hypothesis needs to be confirmed by functional studies.

### The PO transcriptome: candidate genes involved in feeding decisions

Our results showed a relevant compilation of sensory gene families in the PO tissue with the novel finding of *opsins* and *takeouts* (TOs) genes in the gustatory tissue of a blood-sucking insect. This is the first attempt to recognize molecular sensors in an exclusive gustatory tissue of a blood feeder and in particular in a triatomine insect. The current state of knowledge shows that *R. prolixus* engorges when ATP and low salt are present in the feeding solution and rejects it when bitters, high or no salt are present [3]. The underlying mechanisms of these opposing behaviors are still unknown and the primordial step towards their elucidation is to identify the molecular candidates present in the PO.

High expression of *RproGr26*, *RproGr27*, and *RproGr28* was observed in the PO, as was also found in *R. prolixus* antennae previously [66, 67]. The antennal expression pattern of these GRs was high and conserved in nymphs and adults suggesting a putative role in behaviors associated either to host search or interactions with congeners. Finding these highly expressed genes in the PO allows us to contemplate this small set of GRs as major receptors in feeding events. Aversive feeding responses were described with exogenous chemicals for kissing bugs such as bitter compounds [4]. Molecular characterization of the pharyngeal organ of insects is scarce; however, this was performed for *D. melanogaster* in a more recent study in which the expression of bitter and sugar GRs in the pharyngeal GRNs was reported, among other genes [61]. Among the repertoire of expressed GRs of *R. prolixus*, only *RproGr1* shows homology to *DmelGr43a,* which functions as a fructose receptor that triggers satiety responses once high fructose levels are detected [68, 69]. Interestingly, *DmelGr43a*, which is expressed in a few neurons in the protocerebrum, appears to be restricted to some pharyngeal and tarsal GRNs in the fly gustatory system [70]. *R*. *prolixus* is an obligate blood feeder and fructose is not a common component found in blood; however, a recent report of plant feeding in these insects suggests that phytophagy could be an additional feeding habit [71]. Our results reinforce these studies since a fructose sensor could be functional if these hematophagous insects would practice facultative phytophagy as is seen in mosquitoes [20, 72]. Fructose detection, as in the case of bitter detection, could represent the conservation of traits of past ancestors that were either predators or phytophagous [4, 73]. In either case, the feeding response to sugars in kissing bugs remains to be uncovered.

Alternatively, some PO-GRNs may evaluate characteristics other than palatability, such as temperature, humidity, and even viscosity. The most expressed IR in the PO was the *RproIr93a*. This IR is highly conserved among arthropods [37]. In *D. melanogaster*, it was demonstrated to have a role in temperature preference in larvae and moisture detection in the adult antennae [74]. Since evidence shows that this receptor can participate in different mechanisms depending on which IRs are partnered with, functional hypotheses in the pharynx of *R. prolixus* are still indefinite. *RproIr75a* also presented enhanced expressions in the PO. In *D. melanogaster,* antennal *Ir75a* are tuned to acidic molecules, more specifically to acetic acid [75]. The role of *RproIr75a* in the PO needs to be investigated.

PPKs have been described to allow the detection of environmental stimuli like water, salts, or odors [76]. Enhanced expression was observed for the PPK*s Rprc000048*, *Rproppk014276,* and *Rproppk23* in the PO. Recent evidence in a non-feeding context shows that the knockdown of *Rproppk28* and *Rproppk014276* expression through RNA interference leads to a loss of avoidance when walking over substrates with a high concentration of salt [5]. It is plausible then that *Rproppk014276* works as a salt sensor during gustatory assessment in the PO.

TRPs are widely known for multimodal detection of stimuli and also for being highly conserved across different taxons [77, 78]. The PO repertoire of TRPs, although small, represents candidates for vital roles related to heat, water, and noxious compounds detection as was previously demonstrated for other insect models [79–84]. High levels of expression of *RproPainless* and *RproWaterwitch* are observed in the PO as it was previously obtained for the antennal tissue [66, 67]. The current state of knowledge shows that *Painless* and *Trpa1* are necessary to trigger aversive feeding responses to reactive electrophiles, such as allyl isothiocyanate (AITC), a pungent component of horseradish [83, 85, 86]. Moreover, *DmelPainless* intervenes in the avoidance of noxious heat, mechanical stimulation, and dry environments [87–89]. *D. melanogaster* flies detect moist air with their antennae using *Waterwitch* [81]. Likely, *RproPainless* is an appropriate candidate to sense noxious heat and chemicals, while *RproWaterwitch* could fulfill a role in water sensing during feeding in the PO.

The novel finding of opsins in the PO of *R. prolixus* is remarkable since recently it was demonstrated in their role in *D. melanogaster* taste sense [42]. Moreover, it was demonstrated that opsins sense bitter tastants through a signal cascade that also includes a Gq protein, a phospholipase C and a Trpa1. This shows that not only a novel protein is involved in the insect taste sense but also a reminiscent mechanism of mammalian taste transduction such as a GPCR signaling pathway.

High expression of several OBPs, CSPs, and TOs was also found in the PO. Our data support the current knowledge that showed that insect OBPs and CSPs are highly expressed in taste sensilla [90, 91]. Enhanced expression of these auxiliary proteins could indicate a major role during blood feeding. They have been reported to act as solubilizers and carriers of hydrophobic nutrients in the mouthparts of blowflies, moths, and butterflies and as surfactants to reduce pressure during sucking and as detectors of nutrients in moths [92–95]. Starvation seems to regulate the expression of *TO* genes in *D. melanogaster* and food intake was shown to be indirectly affected by this gene family, as was demonstrated for mutant flies [47]. Our results showed that the *TO* family is the most expressed in the PO, suggesting putative feeding regulatory functions in *R. prolixus*.

The high expression of serotonin, acetylcholine, and dopamine receptors suggests relevant functions of these neurotransmitters in the PO functioning. In several insect models, a strong link between biogenic amine levels and modulating hunger/satiety has been demonstrated. Serotonin was demonstrated to modulate appetite, usually depressing feeding in flies, bees, ants, and other insect models [33, 96–99]. In *R. prolixus*, injection of the neurotoxin 5,7-DHT, which depleted serotonin of peripheral neurons, led to a decrease in blood intake [100]. Dopamine activates *DopEcR* in the GRNs to enhance sensitivity to sugar in hungry flies and 20-hydroxyecdysone activates this receptor to repress feeding and promote pupation in lepidopteran insects [54, 55]. The presence of acetylcholine receptors in the alimentary canal is novel and its putative role is unknown. The predominant presence of neurotransmitter receptors in the PO reinforces this organ as a site for the modulation of food acceptance in relation to the internal state and needs of the insect.

The neuroendocrine system regulates feeding behavior and food search in gustatory neural circuits [58]. The *RproGPA2/GPB5* heterodimer was highly expressed, for both the neuropeptide precursor and its receptor, in the PO. The GPA2/GPB5 system was suggested to participate in the control of ion and water balance in the hindgut of *Ae. aegypti* [101]. *RproAst-CC* was the highest expressed NP precursor gene in the PO, as previously found in *R. prolixus* antennae, pointing to a relevant role in the regulation of the perception of chemical stimuli [102]. Other highly expressed precursor genes in the PO were the *RproITG-like*, *RproITP*, *RproMIP*, *RproIDLSRF*-like peptide, and *RproPDF*. The abundance of *RproITG-like* peptides is modulated 24 h post-blood meal [35]. *MIP* reduces the sensitivity toward food in *D. melanogaster*; its silencing causes an increase in food intake and body weight, inducing satiated flies to behave like starved ones [103]. The NPRs with the highest expression in the PO were the *RproFalp/ProcR*, *RproCT/DhR1*, *RproCCAPR1*, *RproCRF/DHR2*, *RproNPLPR*, and *RproInR*. Proctolin stimulates contractions in the midgut and hindgut of insects, suggesting a similar function in the PO of *R. prolixus* [104]. *RproCT/DhR1* is also related to feeding modulation since it increases the contractions of salivary glands and hindgut [105, 106]. Besides, the regulation of blood feeding by the *RproCCAP* system is reinforced by the expression of *RproCCAP* in salivary glands and the *RproCCAP* precursor in projections arriving at the salivary glands [107]. Insects injected with synthetic *RproCRF/DH* peptide before feeding ingested a significantly reduced blood meal [108]. Moreover, *RproNPLP1* is modulated in response to a blood meal in *R. prolixus* [35]. The expression profiles of NPs and NPRs detected in the PO reinforces the hypothesis of these poorly studied neuropeptide systems in regulating *R. prolixus* feeding.

### Misexpression of a PO salt-related sensory gene prevents feeding

Knocking down the expression of PO sensory genes allowed us to establish the functionality of the PO during food assessment. The RNAi knockdown of *Rproppk014276* interferes with feeding acceptance. The role of *Rproppk28* in feeding could not be revealed under the experimental conditions of this work. Certainly, its function in the PO needs further investigation. Beyond the different functions that the large repertoire of genes expressed in the PO can have, our main objective was to establish the crucial role of the PO in food evaluation. Similarly, pharyngeal neurons control food choice and intake in *D. melanogaster,* as was demonstrated in *Poxn* mutant flies [60]. In the case of *R. prolixus,* the PO is the main gustatory organ in the pharynx involved in the evaluation of the incoming food such as blood, but not the antennae nor the tarsi which never come in contact with blood. The gustatory function of the antennae is exclusively related to host skin recognition [4, 5]. Interestingly, *Rproppk014276* modulates the aversive response to high concentration of salt in the antennae during the external skin evaluation [5], whereas it modulates the feeding acceptance to an appetitive solution in the PO during ingestion (this work). Likely, the differential chemosensory context of the antennae (host skin assessment) and the PO (blood evaluation) determines the salience of this gene’s activation.

## Conclusions

The PO, located internally in the initial portion of the alimentary canal, constitutes the final step in food evaluation in *R. prolixus*. Despite their significant relevance across various animal taxa, internal taste organs have been little studied due to difficulties associated with size and physical access. This work demonstrates that the PO of *R. prolixus* contains neurons within the short-peg sensilla, connected to higher brain centers, and expresses genes with the potential function of detecting the different chemical and physical properties of blood. Our characterization of the PO is the primordial step toward elucidating blood acceptance and rejection mechanisms in a non-traditional blood-sucking insect model. This understanding may also pave the way for extrapolation to other blood-sucking models, such as mosquitoes.

## Methods

### Animals

Fifth-instar nymphs and adults of *R. prolixus* were used throughout the experiments. The rearing conditions of insects were 28 °C, ambient relative humidity, and 12-h:12-h light:dark cycle. Insects were kept unfed following ecdysis. Fifth-instar larvae 7–21 days post-ecdysis were used for electrophysiology, feeding assays, and RNA-Seq. Adults used for neuroanatomy experiments were 7–9-day-old post-ecdysis. Feeding experiments were carried out at the beginning of the insects’ scotophase, the moment of the day that the kissing bugs had a maximal motivation to feed [6].

### Neuroanatomy

#### Histology

For the histological analysis of the PO, the heads of insects were fixed for 3 h in a mixture of 2.5% glutaraldehyde and 2.0% paraformaldehyde in phosphate buffer (pH 7.3) with glucose and CaCl_2_. After dehydration, they were embedded via propylene oxide in Durcupan ACM (Electron Microscopy Sciences, Pennsylvania, US). Blocks were serially sectioned at 5–10 μm using glass knives mounted in a microtome. The sections were stained on a hot plate with methylene blue and observed under a light microscope (Olympus, JP).

#### Anterograde fills

Live insects were ventrally fixed to a glass support. An opening was made in the cuticle of the head in the anterodorsal region of the head capsule, in order to reach the somas and axons of the PO-GRNs (Fig. 1A). Subsequently, a drop of distilled water was applied for 6 min. Following this time, the distilled water was absorbed and a drop of the neuronal tracer rhodamine (1% in distilled water, dextran, tetramethylrhodamine, 3000 MW, anionic, lysine fixable, Thermo Fisher, Massachusetts, US) was applied and covered with vaseline to avoid dehydration in a total of 28 adult insects. Then, live insects were maintained inside closed Petri dishes with wet cotton (to assure the maintenance of a humid ambient) for 48 h to allow the neuronal tracer to diffuse to the brain at 8 °C. After this time, the brains were dissected in Millonig’s buffer and fixed in 4% paraformaldehyde overnight at 4 °C. Then, brains were rinsed in Millonig’s buffer, dehydrated through sequential ethanol series and finally cleared and mounted in methyl salicylate [5]. Whole mounts were optically sectioned and scanned with a laser scanning confocal microscope (Olympus FV300/BX61, Centro de Microscopía Avanzada, Facultad de Ciencias Exactas y Naturales, Universidad de Buenos Aires, Buenos Aires, AR).

### Electrophysiological recordings

The summed of action potentials of the PO-GRNs were recorded through extracellular recordings. To access the PO, live insects were fixed dorsally; the proboscis, which is normally folded under the prosternum, was unfolded and fixed. An opening was then made in the cuticle in the anteroventral region of the head capsule, thus exposing the pharynx (Fig. 1A). After the removal of the stylets, the anterior zone of the pharynx was sectioned in half, thus exposing the PO. Note that the PO is usually dry and is only bathed by the incoming blood when the insect feeds. Therefore, when exposing the PO for electrophysiology, the general hemolymph of the body bathes the PO throughout the experiments. The hemolymph, in addition to other ions, is composed of NaCl as one of its main ions. Under these experimental conditions, we used an isosmotic concentration of NaCl (0.15 M) as a control stimulus and to rinse the preparation between stimuli to avoid negative osmotic effects in the tissue and in the living animal. An Ag/AgCl reference electrode was located inside the insect’s abdomen, while a tungsten recording electrode was inserted close to the PO and the labral nerves. Tungsten electrodes were made from wire of diameter 50 μm. Their tips were etched to a point of diameter of 10–15 μm under a dissecting microscope, by passing an alternating current (5 V for 1–3 min) between the tungsten wire and a carbon electrode immersed in saturated KOH. One hundred microliters of each stimulus was applied to the preparation. In a first series of experiments, the following gustatory stimuli applied to the preparation were 0.15 M NaCl, 1 mM ATP (in 0.15 M NaCl), and 5 mM caffeine (in 0.15 M NaCl). Each insect was firstly stimulated with NaCl, followed by stimulation with either ATP or caffeine after 1 min. The preparation was rinsed with 0.15 M NaCl between stimulations. In a second series of experiments, we assessed the dose-dependent effect of NaCl with three doses: 0.05 M, 0.15 M, and 1 M. The interval between subsequent stimulations was 1 min. The biological signals were amplified, filtered (gain × 10, TastePROBE DTP-02, Syntech; gain × 100, eighth-order Bessel, pass-band filter: 10–3000 Hz, Dagan Ex1), digitized (Data Translation DT9803; sampling rate: 10 kHz, 16 bits), and stored in a PC. Spike events detection and firing frequency quantification were performed off-line using dbWave software [109]. We considered positive spike events those whose amplitudes exceeded the baseline noise by two times or more. A 30-point median filter was applied to the signals for better noise discrimination (traces shown in Fig. 1D). To avoid the offset voltage triggered by the addition of the stimulus on the preparation, we used a configurational mode in our acquisition system, in which the initial offset voltage is canceled automatically. ATP was purchased at Sigma-Aldrich (St Louis, US) and caffeine at Biopack (Buenos Aires, AR).

The responses of PO-GRNs to the gustatory stimuli were statistically evaluated using the Kruskal–Wallis test (*α* = 0.05). Subsequently, the responses to ATP and caffeine were compared to the response to NaCl through Dunn’s post hoc comparisons. Nine to 14 biological replicates were carried out in the first series and 10 to 12 in the second series of experiments.

### RNA extraction

The PO is located in the anterodorsal region of the head capsule of *R. prolixus* (Fig. 1A). To isolate the PO, the proboscis was removed first. Then, the anterior part of the head capsule was cut from the insertion of the proboscis in the head capsule to the insertion of the antennae (see light blue square in Fig. 1A). A total of 150 tissue samples were placed in 3 separate tubes (50 POs each) added with RNAlater™ stabilization solution (Invitrogen, Carlsbad, USA) and kept at − 80 °C. Tissue samples were later transferred onto a Whatman™ filter paper Cat N° 1001–055 (Whatman Inc., Florham Park, US) using forceps at room temperature. After absorption of the solution excess, the tissues were immersed in 200 µL of TRIzol® reagent (Invitrogen, Carlsbad, US), macerated with a pestle, and 800 µL of TRIzol was added. The RNA extraction was performed as described in [110]. The total RNA was eluted from the columns with 50 µL of RNase-free water, and an aliquot from each sample was separated for RNA quality check using Agilent 2100 Bioanalyzer (Agilent Technologies, Palo Alto, US). The RNA samples were immediately stored at − 80 °C for further analysis.

### Library preparation and RNA-seq

RNA library preparations and sequencing reactions were conducted at GENEWIZ, LLC. (South Plainfield, US), according to the following protocol. RNA samples were quantified using Qubit 2.0 Fluorometer (Life Technologies, Carlsbad, US) and RNA integrity was checked using Agilent TapeStation 4200 (Agilent Technologies, Palo Alto, US). RNA sequencing libraries were prepared using the NEBNext Ultra RNA Library Prep Kit for Illumina using the manufacturer’s instructions (NEB, Ipswich, US). Briefly, mRNAs were initially enriched with Oligod (T) beads. Enriched mRNAs were fragmented for 15 min at 94 °C. First-strand and second-strand cDNAs were subsequently synthesized. cDNA fragments were end-repaired and adenylated at 3’ ends, and universal adapters were ligated to cDNA fragments, followed by index addition and library enrichment by PCR with limited cycles. The sequencing library was validated on the Agilent TapeStation and quantified by using Qubit 2.0 Fluorometer as well as by quantitative PCR (KAPA Biosystems, Wilmington, US). The sequencing libraries were clustered on a flow cell. After clustering, the flow cell was loaded on the Illumina HiSeq instrument (4000 or equivalent) according to the manufacturer’s instructions. The samples were sequenced using a 2 × 150 bp paired-end configuration. Image analysis and base calling were conducted by the HiSeq Control Software (HCS). Raw sequence data (.bcl files) was converted into fastq files and de-multiplexed using Illumina's bcl2fastq 2.17 software. One mismatch was allowed for index sequence identification. The raw sequence dataset is available in the NCBI BioProject database with the accession number BioProject ID PRJNA674000.

### Bioinformatic analysis

The presence of Illumina sequencing adapters and the quality of reads from the sequencing facility were analyzed using the FASTQC tool (www.bioinformatics.babraham.ac.uk/projects/fastqc). Then, Illumina adapters and those bases from 5’ and 3’ ends with quality scores lower than 5 (TRAILING: 5 and LEADING: 5 parameters) were eliminated from the reads using Trimmomatic v0.32 in the paired-end mode [111]. The SLIDING-WINDOW parameter was fixed at 4:15 and only those reads longer than 50 bp were kept for the next steps. Afterward, trimmed and cleaned reads were mapped and counted to the *R. prolixus* genome assembly (version RproC3.3 from VectorBase) through STAR v.2.6.0 [112] with default parameters and an edited General Feature Format (GFF) file generated by [102]. Raw counts are included in the Additional file 5: Table S1. Heatmaps showing gene expression (expressed as Log_10_(TPM + 1)) of the different protein families were prepared using the gplot package in R [113] (Additional file 5: Table S2). TPM values less than 0.5 were considered as genes not expressed in the PO or below detection limits. TPMs ≥ 0.5 to ≤ 10 is equal to low expression, TPMs ≥ 11 to ≤ 1000 is medium expression, and TPMs > 1000 is equivalent to high expression. However, we cannot rule out that some genes at undetectable levels could be expressed in other moments during the life cycle.

### RNA interference

Double-strand RNA of *Rproppk28* and *Rproppk014276* were synthesized and injected as described in [5]. PCRs were carried out by using specific primers conjugated with 20 bases of the T7 RNA polymerase promoter. The beta-lactamase gene (β-lact) of *Escherichia coli,* an ampicillin resistance gene, was also amplified from the pBluescript SK plasmid as control of dsRNA injection. PCR products for *Rproppk014276*, *Rproppk28*, and β-lact were used as templates for dsRNA synthesis using the MEGAscript™ RNAi Kit (Thermo Fisher Scientific, Massachusetts, US). All PCR fragments were sequenced and checked for their similarity to the expected fragments. After synthesis, the purity and integrity of dsRNA were confirmed through a 1.5% agarose gel and quantified using NanoDrop™ (Thermo Fisher Scientific, Massachusetts, US). The specificity of the dsRNA for each PPK was validated in silico using BLASTn searches against the *R. prolixus* genome sequences, and each dsRNA sequence showed a unique and complete hit against its target sequence.

Insects were randomly separated into four experimental groups. Two groups of insects were injected with the corresponding dsRNAs: dsRNA-Rproppk014276 and dsRNA-Rproppk28. A third group was injected with the dsRNA of β-lact, representing a control for dsRNA injection (dsRNA-ctrl). A microliter syringe (World Precision Instruments, Florida, US) was used to inject 2 μl of dsRNA (concentration = 1.25 µg/µl) diluted in PBS 1 × into the joint between the coxa of the hind leg and the abdomen. The fourth group of insects was maintained intact (uninjected group) as an additional control. Eleven days after dsRNA injection, insects of each group were tested for RT-qPCR verification of gene expression knockdown or behavioral assays (see below).

Knockdown efficacy was assessed in both the antennae, as described in [5], and in the PO. Total RNA was extracted from pools of four to five POs of the dsRNA-ctrl, dsRNA-Rproppk014276, and dsRNA-Rproppk28 groups using the same protocol of [5]. One microgram of extracted RNA was treated with RQ1 RNase-Free DNAse (Promega, Fitchburg, US) to eliminate genomic DNA and afterward used to synthesize cDNA by the M-MLV Reverse Transcriptase system (Promega). qPCR reactions were performed using the StepOnePlus™ Real-time PCR System (Thermo Fisher Scientific, Massachusetts, US) under the following conditions: 10 min at 95 °C, followed by 40 cycles of 20 s at 95 °C, 20 s at 60 °C, and 30 s at 72 °C. After the amplification step, melting curve analysis (HRM rate = 0.5 °C) were performed to confirm reaction specificity. Reactions for each sample were performed in three technical replicates, and no-template controls were included in duplicates for each experiment. The relative gene expression was calculated using the 2 -ΔΔCt method (Pfaffl, 2001). Gene expression in each condition was first normalized by a reference gene (the glutathione S-transferase (GST), Omondi et al., 2015) and then further normalized to the dsRNA-control group. Statistical differences between the transcript levels of the experimental groups were analyzed using the Kruskal–Wallis test, followed by Dunn’s multiple comparisons (Additional file 1: Fig. S1) and Mann–Whitney test (Additional file 2: Fig. S2) (*α* = 0.05).

### Feeding response of dsRNA injected and control insects

The activity of the pharyngeal and cibarial muscles involved in feeding was recorded in dsRNA injected and uninjected insects for 10 min. For this, an artificial feeder coupled to an electromyographic recording system was used [8]. Briefly, the device consists of two containers, the feeder and the insect container. The feeder contains the feeding solution, and the insect container, which is attached to the feeder, holds the insect. The feeder is covered with a clean latex membrane that mimics the host skin, which insects can easily pierce to access the feeding solution. All groups received an appetitive feeding solution—specifically a solution of 1 mM ATP in 0.15 M NaCl (i.e., + NaCl/ + ATP)—as previously defined for *R. prolixus* [8]. Additionally, a control group of uninjected insects was tested with a 1 mM ATP solution in distilled water (i.e., − NaCl/ + ATP), constituting a non-appetitive solution [8]. A metallic mesh connected to a copper wire (recording electrode) was placed inside the insect container. The insect used this metallic mesh to climb and access the feeder. A silver wire (reference electrode) was positioned inside the feeder, in contact with the feeding solution. Both electrodes were connected to a differential amplifier (HotBit HB3600, DE). The experiment started once the insect inserted its mouthparts into the feeder, thereby closing the electrical circuit and generating a base conductance. Changes in the baseline signal were attributed to contractions produced by the sucking muscles when the insect initiated feeding. The recorded signals were amplified (gain × 200) and digitized with aid of the A/D converter of the oscilloscope (Tektronix TDS 210, Oregon, US), which was connected to a PC. Recordings were acquired and analyzed off-line using software designed ad hoc (Diego Anfossi, Héctor Salas Morales, *unpubl.*). The pumping frequency of muscles was calculated by counting the number of pumps (i.e., each pump or peak represented a muscle contraction) during the time the insect sucked the solution. The amplitude of the signals was excluded due to its considerable variation among insects within the same group. Any apparent visual differences were attributed to variations in the electrical contact made by the insect with the recording electrode (metal mesh). The weight gain of insects during feeding was recorded, and a feeding factor was calculated as (Wf − Wi)/Wi, where Wf represents the final weight and Wi the initial weight (Additional files 3 and 4: Fig. S3, S4**)**. Statistical differences among experimental groups were assessed using the Kruskal–Wallis test (*α* = 0.05). Post hoc comparisons were conducted between the uninjected group (fed on + NaCl/ + ATP solution or -NaCl/ +ATP solution) and the other groups using Dunn’s post hoc test. A total of 10 to 19 replicates were performed.

The feeding behavior toward the appetitive solution (+ NaCl/ + ATP) of both antennectomized and intact insects was also assessed in the artificial feeder (Additional file 4: Fig. S4). In this experiment, following the approach outlined in [5], the tips of both antennae (i.e., the distal ends of the second flagellomere) were excised 24 h prior to the experiments. Statistical differences in the weight gain between groups were analyzed using the Mann–Whitney test (*α* = 0.05), with a total of 20 replicates conducted per treatment.

### Supplementary Information

**Additional file 1: Figure S1.** Expression levels of *Rproppk014276* and *Rproppk28* in the antennae after dsRNA injection. Bars represent the relative expression levels (mean ± s.e.m.) of (A) *Rproppk014276* and (B) *Rproppk28*. The transcript levels of *Rproppk014276* and *Rproppk28* in the antennae were significantly decreased compared to the corresponding control groups. Selective knockdown of the gene of interest was also confirmed for both genes (last bars in (A) and (B)). Asterisks indicate significant differences across groups (Kruskal–Wallis test = 13 (in A) and 16.4 (in B), Dunn's post hoc comparisons, *p* < 0.001 in A and B) (*n* = 6 *per* treatment). (A) and (B) Data from Pontes et al. (2022).

**Additional file 2: Figure S2.** Expression levels of *Rproppk014276* in the PO after dsRNA injection. Bars represent the relative expression levels (mean ± s.e.m.) of *Rproppk014276* in dsRNA-Rproppk014276 and dsRNA-ctrl groups. The transcript levels of *Rproppk014276* in the PO were significantly decreased compared to the control group, demonstrating the success of the RNAi knockdown. The asterisk indicates significant differences between the two groups (Mann–Whitney test = 10, *p* = 0.0011) (dsRNA-ctrl, *n* = 12; dsRNA-Rproppk014276, *n* = 8).

**Additional file 3: Figure S3.** Weight gain of treated insects during feeding on the artificial feeder on + NaCl/ + ATP or on the -NaCl/ + ATP solutions. No differences were found between uninjected, dsRNA-ctrl and dsRNA-Rproppk28 groups offered with the + NaCl/ATP solution. dsRNA-Rproppk014276 insects, however, showed significantly lower weight gain than the other groups. The dsRNA-Rproppk014276 group offered with + NaCl/ + ATP solution showed no differences from uninjected insects fed on the -NaCl/ + ATP solution. Scatter plots are shown and bars represent the mean feeding factor (mean ± s.e.m.). The feeding factor was calculated as a normalized weight gain as follows (Wf—Wi)/Wi; where Wf: final weight, Wi: initial weight. Asterisks indicate significant differences of each group to uninjected insects fed on the + NaCl/ + ATP solution (Kruskal–Wallis test = 26.6, *p* < 0.0001, Dunn's post hoc comparisons, p < 0.05) (+ NaCl/ + ATP groups: uninjected, *n* = 14; dsRNA-ctrl, *n* = 15; dsRNA-Rproppk28, *n* = 19; dsRNA-Rproppk014276, *n* = 13; and -NaCl/ + ATP uninjected group, *n* = 10).

**Additional file 4: Figure S4.** Weight gain of antennectomized and intact insects during feeding on the artificial feeder with + NaCl/ + ATP. Only the tips of the last flagellomeres of both antennae, where salt detectors are localized (Pontes et al. 2022), were excised in antennectomized insects. Feeding acceptance of both groups was similar (Mann–Whitney test = 184.5, *p* = 0.341), emphasizing that *R. prolixus* does not use the antennae to detect NaCl present in the feeding solution. Scatter plots are shown and bars represent the mean feeding factor (mean ± s.e.m.). The feeding factor was calculated as the normalized weight gain using the formula (Wf—Wi)/Wi; where Wf represents the final weight and Wi represents the initial weight (*n* = 20 *per* treatment).

**Additional file 5: Table S1.** Raw count matrix generated by STAR after mapping trimmed and cleaned reads against the *R. prolixus* genome. **Table S2.** Transcripts *per* kilobase *per* Million read (TPM) values in the three replicates for all targeted genes. TPM values > 1 are in light blue.

## Data Availability

All data generated or analyzed during this study are included in this published article, its supplementary information files, and publicly available repositories. Behavioral, electrophysiological raw data and images are deposited on Mendeley Data (doi: 10.17632/bpgj85yfs6.1). Sequencing data is available at https://www.ncbi.nlm.nih.gov/sra/?term=PRJNA674000. Any additional information is available from the lead contact upon request.

## Abbreviations

PO: Pharyngeal organ
PO-GRNs: Pharyngeal organ gustatory receptor neurons
SEZ: Subesophageal zone
CNS: Central nervous system
ds-RNA: Double-stranded RNA
TPM: Transcripts per kilobase per million read
GRs: Gustatory receptors
IRs: Ionotropic receptors
PPKs: Pickpocket channels
TRPs: Transient receptor potential channels;
Ops: Opsins
OBPs: Odorant-binding proteins
CSPs: Chemosensory proteins
TOs: Takeouts
NPs: Neuropeptide precursors
NPRs: Neuropeptide precursor receptors
BARs: Biogenic amines receptors
