# Supplementary figures and images for: The pharyngeal taste organ of a blood-feeding insect functions in food recognition
Source: BMC Biol. 2024 Mar 13;22:63. doi: 10.1186/s12915-024-01861-w (PMC10938694; doi:10.1186/s12915-024-01861-w)

Figure S1

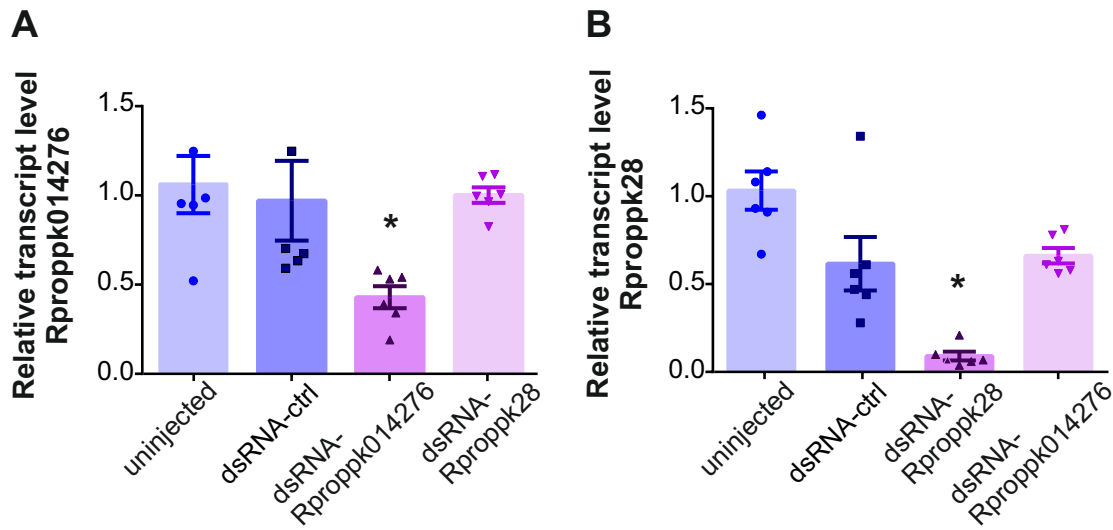

Supplement: Supplementary file 1 — Additional file 1: Figure S1. Expression levels of Rproppk014276 and Rproppk28 in the antennae after dsRNA injection. Bars represent the relative expression levels (mean ± s.e.m.) of (A) Rproppk014276 and (B) Rproppk28. The transcript levels of Rproppk014276 and Rproppk28 in the antennae were significantly decreased compared to the corresponding control groups. Selective knockdown of the gene of interest was also confirmed for both genes (last bars in (A) and (B)). Asterisks indicate significant differences across groups (Kruskal–Wallis test = 13 (in A) and 16.4 (in B), Dunn's post hoc comparisons, p < 0.001 in A and B) (n = 6 per treatment). (A) and (B) Data from Pontes et al. (2022). [file 12915_2024_1861_MOESM1_ESM.pdf]

Figure S2

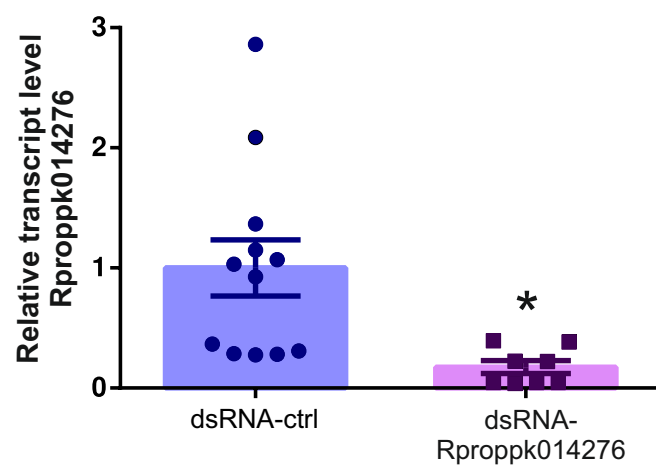

Supplement: Supplementary file 2 — Additional file 2: Figure S2. Expression levels of Rproppk014276 in the PO after dsRNA injection. Bars represent the relative expression levels (mean ± s.e.m.) of Rproppk014276 in dsRNA-Rproppk014276 and dsRNA-ctrl groups. The transcript levels of Rproppk014276 in the PO were significantly decreased compared to the control group, demonstrating the success of the RNAi knockdown. The asterisk indicates significant differences between the two groups (Mann–Whitney test = 10, p = 0.0011) (dsRNA-ctrl, n = 12; dsRNA-Rproppk014276, n = 8). [file 12915_2024_1861_MOESM2_ESM.pdf]

Figure S3

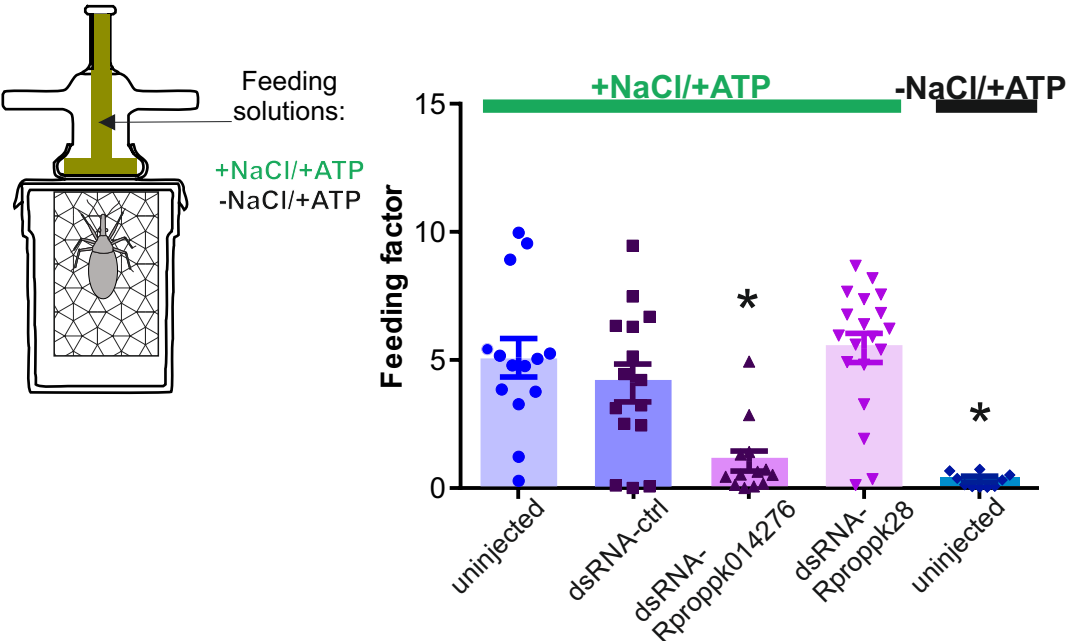

Supplement: Supplementary file 3 — Additional file 3: Figure S3. Weight gain of treated insects during feeding on the artificial feeder on + NaCl/ + ATP or on the -NaCl/ + ATP solutions. No differences were found between uninjected, dsRNA-ctrl and dsRNA-Rproppk28 groups offered with the + NaCl/ATP solution. dsRNA-Rproppk014276 insects, however, showed significantly lower weight gain than the other groups. The dsRNA-Rproppk014276 group offered with + NaCl/ + ATP solution showed no differences from uninjected insects fed on the -NaCl/ + ATP solution. Scatter plots are shown and bars represent the mean feeding factor (mean ± s.e.m.). The feeding factor was calculated as a normalized weight gain as follows (Wf—Wi)/Wi; where Wf: final weight, Wi: initial weight. Asterisks indicate significant differences of each group to uninjected insects fed on the + NaCl/ + ATP solution (Kruskal–Wallis test = 26.6, p < 0.0001, Dunn's post hoc comparisons, p < 0.05) (+ NaCl/ + ATP groups: uninjected, n = 14; dsRNA-ctrl, n = 15; dsRNA-Rproppk28, n = 19; dsRNA-Rproppk014276, n = 13; and -NaCl/ + ATP uninjected group, n = 10). [file 12915_2024_1861_MOESM3_ESM.pdf]

Feeding solution:  
+NaCl/+ATP

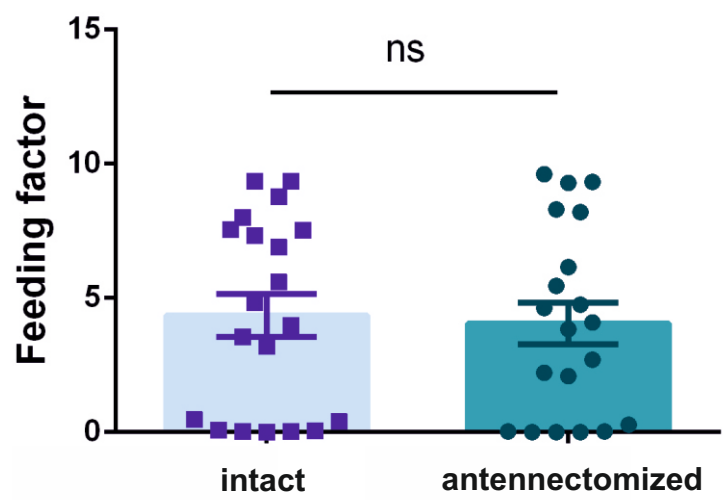

Supplement: Supplementary file 4 — Additional file 4: Figure S4. Weight gain of antennectomized and intact insects during feeding on the artificial feeder with + NaCl/ + ATP. Only the tips of the last flagellomeres of both antennae, where salt detectors are localized (Pontes et al. 2022), were excised in antennectomized insects. Feeding acceptance of both groups was similar (Mann–Whitney test = 184.5, p = 0.341), emphasizing that R. prolixus does not use the antennae to detect NaCl present in the feeding solution. Scatter plots are shown and bars represent the mean feeding factor (mean ± s.e.m.). The feeding factor was calculated as the normalized weight gain using the formula (Wf—Wi)/Wi; where Wf represents the final weight and Wi represents the initial weight (n = 20 per treatment). [file 12915_2024_1861_MOESM4_ESM.pdf]
